# Residue contacts predicted by evolutionary covariance extend the application of *ab initio* molecular replacement to larger and more challenging protein folds

**DOI:** 10.1107/S2052252516008113

**Published:** 2016-06-15

**Authors:** Felix Simkovic, Jens M. H. Thomas, Ronan M. Keegan, Martyn D. Winn, Olga Mayans, Daniel J. Rigden

**Affiliations:** a Institute of Integrative Biology, University of Liverpool, Liverpool L69 7ZB, England; b Research Complex at Harwell, STFC Rutherford Appleton Laboratory, Didcot OX11 0FA, England; c Science and Technology Facilities Council, Daresbury Laboratory, Warrington WA4 4AD, England

**Keywords:** molecular replacement, protein structure prediction, evolutionary covariation, predicted contacts, *ab initio* modelling

## Abstract

For many protein families, the deluge of new sequence information together with new statistical protocols now allow the accurate prediction of contacting residues from sequence information alone. This offers the possibility of more accurate *ab initio* (non-homology-based) structure prediction. Such models can be used in structure solution by molecular replacement (MR) where the target fold is novel or is only distantly related to known structures. Here, *AMPLE*, an MR pipeline that assembles search-model ensembles from *ab initio* structure predictions (‘decoys’), is employed to assess the value of contact-assisted *ab initio* models to the crystallographer. It is demonstrated that evolutionary covariance-derived residue–residue contact predictions improve the quality of *ab initio* models and, consequently, the success rate of MR using search models derived from them. For targets containing β-structure, decoy quality and MR performance were further improved by the use of a β-strand contact-filtering protocol. Such contact-guided decoys achieved 14 structure solutions from 21 attempted protein targets, compared with nine for simple *Rosetta* decoys. Previously encountered limitations were superseded in two key respects. Firstly, much larger targets of up to 221 residues in length were solved, which is far larger than the previously benchmarked threshold of 120 residues. Secondly, contact-guided decoys significantly improved success with β-sheet-rich proteins. Overall, the improved performance of contact-guided decoys suggests that MR is now applicable to a significantly wider range of protein targets than were previously tractable, and points to a direct benefit to structural biology from the recent remarkable advances in sequencing.

## Abstract 2

Residue-contact predictions extend the range of *ab initio* molecular replacement.

## 1. Introduction   

Molecular replacement (MR) is the most common technique for deriving the lost phase information of the unknown target structure in X-ray crystallography. MR places a structurally similar protein in the unit cell of the unknown target to best reproduce the diffraction data. The correct placement of the similar structure provides the basis for the initial phase calculation of the target. The obtained phasing information and the measured diffraction intensities allow the initial calculation of the electron-density map (Blow & Rossmann, 1961 ▸). However, homologues of the target structure do not always exist or may be too structurally distinct. On the other hand, experimental alternatives to MR such as anomalous dispersion (Matthews, 1966 ▸; Hendrickson *et al.*, 1985 ▸; Wang, 1985 ▸) or isomorphous replacement (Green *et al.*, 1954 ▸; Perutz, 1956 ▸; Blow & Rossmann, 1961 ▸) can be time-consuming or difficult to implement in certain cases. These considerations have driven recent developments in computational crystallo­graphy to derive and trial search models from unconventional sources. Such sources include ideal secondary-structure elements or structural motifs (Rodríguez *et al.*, 2009 ▸), libraries of tertiary structural cores derived from mining the Protein Data Bank (PDB; Sammito *et al.*, 2013 ▸) and *ab initio* protein structure predictions (‘decoys’; Qian *et al.*, 2007 ▸; Rigden *et al.*, 2008 ▸; Das & Baker, 2009 ▸; Bibby *et al.*, 2012 ▸; Keegan *et al.*, 2015 ▸; Rämisch *et al.*, 2015 ▸; Thomas *et al.*, 2015 ▸). Clearly, the success of approaches based on *ab initio* protein modelling will depend sensitively on the quality of the structure predictions available.

In recent years, a step change in the accuracy of residue–residue contact predictions (Giraud *et al.*, 1999 ▸; Miller & Eisenberg, 2008 ▸; Weigt *et al.*, 2009 ▸; Burger & van Nimwegen, 2010 ▸), based on sequence information alone, has enabled striking advances to be made in structural bioinformatics, including in *ab initio* modelling. Although evolutionary covariance analysis for contact prediction is a research area with a long history (Levitt & Warshel, 1975 ▸; Vendruscolo *et al.*, 1997 ▸), only recently has the prediction of such contacts become sufficiently accurate to guide *ab initio* structure prediction successfully (Supplementary Fig. S1; Marks *et al.*, 2011 ▸; Kosciolek & Jones, 2014 ▸; Michel *et al.*, 2014 ▸; Adhikari *et al.*, 2015 ▸; Ovchinnikov *et al.*, 2015 ▸). The basic rationale behind the prediction of residue–residue contacts rests on the existence of strong evolutionary constraints, reflected in the covariation of contacting residues, to maintain functionally important conformations. Such evolutionary constraints can be detected at a sequence level, but thousands of homologous protein sequences are required to detect them. The great challenge of separating direct and indirect residue–residue contacts (direct, A–B and B–C; indirect, A–C) was recently overcome through the use of cooperative (‘global’) statistical probability models. These approaches not only treat contact pairs independently, but analyse their dependence on each other, thereby increasing the signal-to-noise ratio in the predicted list of contacts or ‘contact map’ (Giraud *et al.*, 1999 ▸; Miller & Eisenberg, 2008 ▸; Weigt *et al.*, 2009 ▸; Marks *et al.*, 2011 ▸). Therefore, contacts with the strongest signal, indicated by the highest global statistical scores, are most likely to represent the true residue interactions in a protein conformation (Marks *et al.*, 2012 ▸).

Since the successful separation of direct and indirect contacts, various evolutionary covariance-analysis applications have been developed to increase the accuracy and speed of contact predictions (Balakrishnan *et al.*, 2011 ▸; Jeong & Kim, 2012 ▸; Jones *et al.*, 2012 ▸; Ekeberg *et al.*, 2013 ▸, 2014 ▸; Kamisetty *et al.*, 2013 ▸; Wang & Xu, 2013 ▸; Feinauer *et al.*, 2014 ▸; Kaján *et al.*, 2014 ▸; Schneider & Brock, 2014 ▸; Seemayer *et al.*, 2014 ▸; Skwark *et al.*, 2014 ▸). Broadly, these applications can be divided into three categories depending on the cooperative statistical model implemented to derive evolutionary covariance amongst multiple homologous sequences. The first category employs a pseudo-likelihood maximization model and can be found in applications such as *plmDCA* (Ekeberg *et al.*, 2013 ▸; Kamisetty *et al.*, 2013 ▸), *GREMLIN* (Kamisetty *et al.*, 2013 ▸) or *CCMpred* (Seemayer *et al.*, 2014 ▸). The second and third categories include sparse covariance matrix inversion models such as *PSICOV* (Jones *et al.*, 2012 ▸) or mean-field direct coupling analysis models such as *EVFold-mfDCA* (Kaján *et al.*, 2014 ▸), respectively. Although these methods differ in accuracy and speed, a recent study (Jones *et al.*, 2015 ▸) revealed a high similarity of around 90% in top-ranked contacts amongst those three categories. To capture the full spectrum of top-ranked contacts and produce the best possible contact map, metapredictors such as *PconsC*2 (Skwark *et al.*, 2013 ▸, 2014 ▸) or *MetaPSICOV* (Jones *et al.*, 2015 ▸) combine individual contact predictions across two or three of these categories. Additionally, an ongoing aim of prediction tools is to achieve successful detection of evolutionary covariance from homologous sequence sets of reduced diversity and number. Any decrease in the number of homologous sequences required would make covariance analysis applicable to smaller protein families.

One of the principal applications of predicted contact maps is to predict structures for large protein families (Marks *et al.*, 2011 ▸; Kosciolek & Jones, 2014 ▸; Michel *et al.*, 2014 ▸; Adhikari *et al.*, 2015 ▸; Ovchinnikov *et al.*, 2015 ▸). Without contact information, accurate homology-independent fold predictions for globular proteins in *Rosetta* are limited to chain lengths of up to ∼130 residues (Kinch *et al.*, 2011 ▸; He *et al.*, 2013 ▸; Tai *et al.*, 2014 ▸). Several covariance analysis tools, such as *GREMLIN* (Ovchinnikov *et al.*, 2015 ▸), *PconsC*2 (Michel *et al.*, 2014 ▸; Skwark *et al.*, 2014 ▸), *MetaPSICO*V (Jones *et al.*, 2015 ▸; Kosciolek & Jones, 2015 ▸) and *EVFold* (Marks *et al.*, 2011 ▸), reported accurate fold predictions for much larger globular proteins, illustrating how the use of contact predictions can greatly expand the capabilities of *ab initio* folding protocols.

The availability of improved tertiary-structure predictions from contact-assisted fragment-assembly *ab initio* modelling naturally enhances the prospects for their use in MR. Broadly speaking, two approaches have been reported. The first entails highly CPU-intensive modelling to produce an overall fold prediction that is sufficiently accurate to serve as a search model in the same way as a crystal structure or homology model would conventionally be deployed (Qian *et al.*, 2007 ▸; Das & Baker, 2009 ▸). Alternatively, more cheaply obtained, coarse-grained models can be clustered into search-model ensembles and, recognizing their limited accuracy, treated to truncation to attempt to isolate sufficiently accurate core regions (Rigden *et al.*, 2008 ▸; Bibby *et al.*, 2012 ▸). *AMPLE* (***a**b initio* **m**odelling of **p**roteins for mo**le**cular replacement; Bibby *et al.*, 2012 ▸) is a pipeline that implements the latter strategy and is available in the *CCP*4 software suite (Winn *et al.*, 2011 ▸). *AMPLE* overcomes the absence of suitable crystal structures or homology models through a cluster-and-truncate approach that processes computationally cheap and minimally refined *Rosetta* (Bibby *et al.*, 2012 ▸) or *QUARK* (Keegan *et al.*, 2015 ▸) *ab initio* decoys into search models. In brief, 1000 *ab initio* decoys are clustered based on their structural similarity, after which the decoys in the largest resulting cluster (containing a maximum of 200 decoys) are truncated at 20 different intervals. Truncation is rationally guided by inter-decoy structural variance within the cluster (Qian *et al.*, 2007 ▸; Bibby *et al.*, 2012 ▸). The truncated decoys are then subclustered under three different C^α^ r.m.s.d. radii (1, 2 and 3 Å), whereby a maximum of 30 decoys within the cluster, those closest to the cluster centroid, are selected and combined into an ensemble. Lastly, each ensemble search model undergoes three different side-chain treatments: polyalanine (all side chains are truncated at their C^β^ atom), reliable side chains (only side chains are kept that are usually well modelled; Shapovalov & Dunbrack, 2007 ▸) and all-atom (all side chains are kept). Up to 120 ensemble search models can be obtained per target through this cluster-and-truncate approach, but this number strongly depends on the structural similarity of the initial decoys and the similarity of the decoys after truncation. Each ensemble search model is processed using *MrBUMP* (Keegan & Winn, 2008 ▸), which in turn uses *Phaser* (McCoy *et al.*, 2007 ▸) and/or *MOLREP* (Vagin & Teplyakov, 1997 ▸, 2010 ▸) for MR, *SHELXE* (Sheldrick, 2010 ▸; Thorn & Sheldrick, 2013 ▸) for main-chain tracing and *ARP*/*wARP* (Cohen *et al.*, 2008 ▸) or *Buccaneer* (Cowtan, 2006 ▸) for automatic rebuilding of the *SHELXE* trace.

In an initial study of 295 small globular proteins with fewer than 120 residues and resolution better than 2.2 Å, 43% of the targets were solved successfully (Bibby *et al.*, 2012 ▸). However, the application of *ab initio* modelling to MR, and therefore the success of *AMPLE*, is greatly limited by the size and fold class of the protein target. These limitations arise at the initial stage during decoy prediction, where successful fold predictions of protein structures without homologues of known structure are currently limited to a chain length of ∼130 residues (Kinch *et al.*, 2011 ▸; He *et al.*, 2013 ▸; Tai *et al.*, 2014 ▸). *AMPLE* has been shown to succeed with protein targets greater than 150 amino acids in size, but these cases were not comparable in the nature of the target and/or the methodology employed: the successes were achieved with either anisometric folds (*e.g.* coiled coils), ensemble search models derived from distant structural homologues or NMR structures (Bibby *et al.*, 2013 ▸; Bruhn *et al.*, 2014 ▸; Hotta *et al.*, 2014 ▸; Thomas *et al.*, 2015 ▸). Thus, the largest globular protein target previously solved with *AMPLE* using *ab initio* models is 120 amino acids in length (Bibby *et al.*, 2012 ▸), although it was noted in that work that success rates had not declined to zero at this size threshold. In addition to the issue of protein size, the success rate of *AMPLE* strongly depends on the fold architecture, as reflected in the widely varying success rates of all-α (80%), mixed α–β (including α/β and α+β folds; 37%) and all-β protein targets (2%) in the original test set of 295 small globular proteins. In sum, both the size and the fold of the target can limit *ab initio* folding protocols and thus the success rate of *AMPLE*.

*Ab initio* modelling of proteins without exploiting information from known folds is a longstanding challenge in the field of computational structural biology and success currently strongly depends on the chain length and fold architecture of the target protein. Recent successful advances in the derivation of direct residue–residue contacts from large multiple sequence alignments have greatly increased the accuracy of *ab initio* structure predictions, especially for larger and all-β protein targets, which are the greatest challenges for *ab initio*-based MR approaches. Here, we set out to explore the impact of the improved contact-guided decoys on the success rate of MR. For this, we use our automated pipeline *AMPLE*, as its cluster-and-truncate approach has proven to be highly successful in the downstream processing of *ab initio* decoys for MR (Bibby *et al.*, 2012 ▸). We report that contact-guided decoys allow the successful solution of targets that were previously unsolvable using the *AMPLE* method. In addition, we report that combining independently obtained contact maps further improves decoy quality, which in turn extends the tractable MR target range to β-rich proteins.

## 2. Methods   

### 2.1. Data set   

A test set of 21 globular protein targets was used throughout. They were manually selected to include a range of chain lengths, fold architectures, X-ray diffraction data resolutions and divergent sequence counts in a multiple sequence alignment. The test set covered the three fold classes (α-helical, mixed α–β and β-sheet) and each target was grouped based on its secondary-structure content as defined by *DSSP* (Kabsch & Sander, 1983 ▸; Joosten *et al.*, 2011 ▸; Supplementary Table S1). The chain length of the sequences ranged from 62 to 221 residues and each crystal structure contained one molecule in the asymmetric unit. The resolutions of the crystal structures ranged from 1.0 to 2.3 Å. The FASTA sequences of each target, as provided in the PDB entry (Rose *et al.*, 2015 ▸), were modelled, rather than the sequence that was visibly present in the crystallographic model. A number of divergent (‘effective’) sequences (*N*_eff_) available for a target of greater than 100 is considered to be the minimum requirement for accurate covariance-based contact predictions (Skwark *et al.*, 2014 ▸). The formula *N*_eff_ = 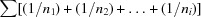 (Jones *et al.*, 2015 ▸) defines *N*_eff_ as the sum of fractional weights of *n* sequences in *i* clusters in a multiple sequence alignment (MSA). To calculate this parameter for our targets, each target sequence formed the basis of an MSA which was obtained from a database search with *HHblits* v.2.0.15 (Remmert *et al.*, 2012 ▸). Two sequence-search iterations were performed with an *E*-value cutoff of 10^−3^ against the nonredundant UniProt20 database v.2013.03 (The UniProt Consortium, 2015 ▸). All sequences in each resulting alignment were then clustered using *CD-HIT* v.4.6.3 (Li *et al.*, 2001 ▸, 2002 ▸; Fu *et al.*, 2012 ▸) at 62% sequence identity (Jones *et al.*, 2015 ▸) and *N*_eff_ was calculated.

### 2.2. Evolutionary covariance analysis   

One contact map was predicted for each of the 21 targets using the fully automated metapredictor *PconsC*2 (Skwark *et al.*, 2014 ▸). In summary, MSAs were generated with *Jackhmmer* (Johnson *et al.*, 2010 ▸) against the UniRef100 database and with *HHblits* v.2.0.15 (Remmert *et al.*, 2012 ▸) against the non­redundant UniProt20 database v.2013.03 (The UniProt Consortium, 2015 ▸) at *E*-value cutoffs of 10^−40^, 10^−10^, 10^−4^ and 1. Each MSA was then analysed with *PSICOV* (Jones *et al.*, 2012 ▸) and *plmDCA* (Ekeberg *et al.*, 2013 ▸, 2014 ▸) to produce 16 individual sets of contact predictions. All 16 predictions, combined with a secondary-structure prediction, solvent-accessibility information and a sequence profile, were then provided to a deep-learning algorithm (Skwark *et al.*, 2014 ▸) to identify protein-like contact patterns. The latter produced a final contact map for each target sequence.

An additional contact map for β-structure-containing targets was predicted using *CCMpred* (Seemayer *et al.*, 2014 ▸) and reduced to β-sheet contact pairs using the *CCMpred*-specific filtering protocol *bbcontacts* (Andreani & Söding, 2015 ▸). Each MSA for *CCMpred* contact predictions was obtained using *HHblits* v.2.0.15 (Remmert *et al.*, 2012 ▸). This entailed two sequence-search iterations with an *E*-value cutoff of 10^−3^ against the nonredundant UniProt20 database v.2013.03 (The UniProt Consortium, 2015 ▸) and filtering to 90% sequence identity using *HHfilter* v.2.0.15 (Remmert *et al.*, 2012 ▸) to reduce sequence redundancy in the MSA. Besides the contact matrix as input, *bbcontacts* requires a secondary-structure prediction and a factor describing the range of predicted contacts in the MSA. The latter was shown to depend on the sequence count in the MSA (*N*) and the target chain length (*L*). Thus, the factor describing this MSA-specific diversity was calculated using the equation η = (*N*/*L*)^1/2^ (Andreani & Söding, 2015 ▸). The secondary structure for each sequence was predicted using the addss.pl (Remmert *et al.*, 2012 ▸) script distributed with *HH-suite* v.2.0.16 (Söding, 2005 ▸). Hereafter, the term *bbcontacts* will be used to describe the full process from the target sequence to the filtered β-strand contact map. At no point do contact-prediction algorithms use structural information from structurally characterized proteins.

### 2.3. Conversion of contact maps to contact restraints   

For all targets, the predicted contact maps from *PconsC*2 were converted to *Rosetta* (Rohl *et al.*, 2004 ▸) restraints to guide *ab initio* folding of the target sequences. The *FADE* energy function was used to introduce a restraint in the folding protocol of *Rosetta*. As described in *PconsFold* (Michel *et al.*, 2014 ▸), a restraint was satisfied during folding if the participating C^β^ atoms (C^α^ in the case of glycine) were within 9 Å of one another. If a pre-defined contact restraint was satisfied, a smoothed ‘squared-well’ bonus was added to the internal energy scoring function of *Rosetta* during folding. The shape of this function therefore rewards conformations that place residues within 9 Å of each other, but has no influence on the energy outside this range. Thus, a false-positive prediction between two positions that are in fact distant in the target structure will not lead to an undesirable long-distance attraction between the two residues. As defined by Michel *et al.* (2014 ▸), the ‘squared-well’ bonus (parameter wd in *FADE*) was set to −15.00. Adopting the same benchmarked approach as Michel *et al.* (2014 ▸), only the top *L* ranked contacts (based on confidence scores, with *L* again representing target length) from each *PconsC*2 contact map were selected and converted to *Rosetta* restraints.

For β-containing targets, an alternative selection of predicted contacts, hereafter called *PconsC*2+*bbcontacts*, was made by a novel combination of *PconsC*2 and *bbcontacts* predictions, as follows. Firstly, inter-strand predictions composed of only one or two contacts were removed from the *bbcontacts* contact list owing to their high false-positive rate (Jessica Andreani, personal communication). For all present contact pairs between residues *i* and *j* and any neighbouring contacts (*i.e.* *i*, *j* ± 1; *i*, *j* ± 2; *i* ± 1, *j*; *i* ± 2, *j*) in the top-*L* *PconsC*2 contact list the ‘squared-well’ bonus was doubled from −15.00 to −30.00, which proved to be the most effective after several options were tried (unpublished data). In addition, all contact pairs solely present in the filtered *bbcontacts* contact map were added to the modified *PconsC*2 and *bbcontacts* contact list with a ‘squared-well’ bonus of −15.00. It is worth noting that the added *bbcontacts* contacts were also present in the full *PconsC*2 contact map, although they were not within the top-*L* cutoff. This approach allowed a strengthening of the weight on β-strand contacts during *ab initio* structure prediction. After uniting the two predictions in this way no further length-based cutoff was applied, so that the *PconsC*2+*bbcontacts* restraint list fed to *Rosetta* for β-containing proteins might be longer than the simple *PconsC*2 list.

The final contact-prediction lists were compared with the corresponding crystal structure contacts to determine their accuracy. For this, all pairs of C^β^ atoms (C^α^ in the case of glycine) within 9 Å of one another in the crystal structure were considered as reference contacts. Predictions were assigned as true or false positives according to whether they were in the list of reference contacts or not. The precision or positive predictive value (PPV) for the each restraint list was then determined using the formula PPV = (true positives)/(true positives + false positives).

### 2.4. *Ab initio* structure prediction of decoys   

Fragments were picked for unassisted *Rosetta* modelling with secondary-structure prediction from *PSIPRED* (McGuffin *et al.*, 2000 ▸) and for contact-assisted decoys with the secondary-structure prediction obtained during evolutionary covariance analysis. Homologous structures were excluded using the nohoms flag to make all experiments equivalent to predictions of unknown folds. Protein decoys were generated using the *AbinitioRelax* folding protocol of *Rosetta* in v.2015wk05 (Rohl *et al.*, 2004 ▸). As recommended in the *Rosetta* documentation, special modelling parameters included the helix and loop atom-refinement flags abinitio::rsd_wt_helix and abinitio::rsd_wt_loop with a reweight factor of 0.5. The two flags abinitio:relax and relax::fast were set during *ab initio* modelling to obtain an all-atom refinement using the *Rosetta* full-atom force field. For each target in the data set, the structure folds were predicted under two different restraint conditions: without any residue–residue contact restraints and with *PconsC*2-only contact restraints. Targets containing β-folds were additionally modelled with a third restraint condition: *PconsC*2+*bbcontacts* contact restraints derived as described above. *bbcontacts*-only contact restraints were not treated as a separate condition owing to the low count of predicted contacts. A total of 1000 decoys were modelled for each target under each of the three different restraint conditions. Decoy quality was assessed based on the template-modelling score (TM-score; Zhang & Skolnick, 2005 ▸), a measure of fold similarity between two structures with identical sequences, in this case a decoy and the corresponding crystal structure. TM-scores range from 0 to 1, with a TM-score above 0.5 usually indicating a correct fold prediction.

### 2.5. Molecular replacement   

The three sets of *ab initio* decoys for each target were subjected to *SCWRL*4 side-chain remodelling (Canutescu *et al.*, 2003 ▸; Krivov *et al.*, 2009 ▸). Afterwards, all three sets of decoys for each target were run in the automated MR pipeline *AMPLE* v.1.0 using default parameters, with the exception of the number of clusters to trial, which was changed from one to three (Bibby *et al.*, 2012 ▸). The associated structure-factor amplitudes for the crystal structure of each target were retrieved from the PDB. The correct placement of search models by *Phaser* was assessed using the recently developed residue-independent overlap (RIO) score (Thomas *et al.*, 2015 ▸). In short, the RIO score assesses the in-sequence and out-of-sequence register overlap of the placed search-model residues (fragments of at least three residues) with the corresponding crystal structure. To be considered a success, MR using *AMPLE* was required to give a *SHELXE* correlation coefficient (CC) of ≥25.00 and an average chain length (ACL) of ≥10.00 (Sheldrick, 2010 ▸; Thorn & Sheldrick, 2013 ▸; Keegan *et al.*, 2015 ▸). Additionally, structure rebuilding of the *SHELXE* chain traces was attempted using both *ARP*/*wARP* (Cohen *et al.*, 2008 ▸) and *Buccaneer* (Cowtan, 2006 ▸) and an *R*_free_ value of ≤0.45 from either method was required.

## 3. Results   

### 3.1. Both general and β-strand-specific residue–residue contact maps improve *ab initio* protein structure predictions   

The initial part of this study evaluates the use of contact restraints for improving *ab initio* protein structure prediction. For each protein target in the data set, 1000 decoys were predicted using *Rosetta*, either unassisted or with restraints deriving from *PconsC*2 alone or from our novel fusion of *PconsC*2 and *bbcontacts* predictions. Since *AMPLE* by default processes decoys from the largest cluster to create search models, structure quality was primarily assessed for these structure predictions alone. However, we also report overall improvements for all decoys (Table 1 ▸).

A high degree of sequence diversity is a prerequisite for the identification of residue covariance in MSAs. The range of effective sequences in the MSAs of target sequences ranged from 272 to 1831. Typically, higher numbers of effective sequences correlate with more accurate contact predictions (Jones *et al.*, 2012 ▸; Kamisetty *et al.*, 2013 ▸; Ekeberg *et al.*, 2014 ▸; Skwark *et al.*, 2014 ▸; Ma *et al.*, 2015 ▸). Here, similar results were observed, as illustrated in Supplementary Fig. S2. Considering the three fold classes, more accurate predictions were obtained for β-structure-containing proteins (median PPV_β_ = 0.940; median PPV_α–β_ = 0.909) compared with all-α targets (median PPV_α_ = 0.655). In plain language, for β-structure-containing proteins over 90% of the intramolecular contacts predicted by evolutionary covariance methods are indeed present in the crystal structure. Although untested in this study, a higher accuracy for β-sheet-containing proteins is achieved owing to the regular pattern of contact pairs that is easily detectable in a contact map (Skwark *et al.*, 2014 ▸; Andreani & Söding, 2015 ▸). The deep-learning procedure used during the final step of *PconsC*2 filters these contact pairs better, therefore increasing the overall accuracy of the prediction.

**Figure 1:**
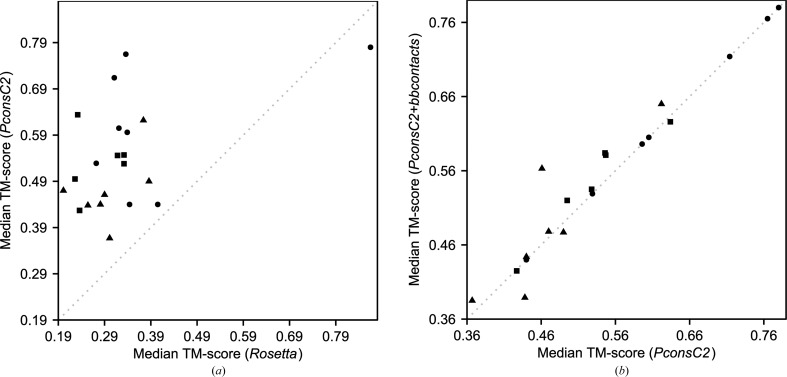
Residue–residue contact restraints improve *ab initio* model quality. (*a*) Median template-modelling scores (TM-scores) for *Rosetta* decoys plotted against median TM-scores for *PconsC*2-only coupling-guided decoys. (*b*) Median TM-score for *PconsC*2-only coupling-guided decoys plotted against median TM-scores for *PconsC*2+*bbcontacts* decoys (Skwark *et al.*, 2014 ▸; Andreani & Söding, 2015 ▸). Median TM-scores derived from decoys found in the largest cluster. The symbol shapes correspond to the three different fold classes: all-α (circles), all-β (triangles) and mixed α–β (squares).

As expected (Michel *et al.*, 2014 ▸; Skwark *et al.*, 2014 ▸), the inclusion of *PconsC*2-predicted contact information substantially improved the quality of structure predictions. A simple *Rosetta* run without contact information yielded a largest cluster median TM-score of 0.342 for all 21 protein targets, compared with 0.542 for *PconsC*2-only decoys (Fig. 1 ▸ *a*). 20 of 21 targets were modelled better, with median TM-score improvements ranging from 0.035 to 0.429. A single target, PDB entry 2qyj, the unassisted *Rosetta* models for which were already of exceptionally high quality (median TM-score of largest cluster structure predictions of 0.865), was modelled slightly worse (0.780) when contact information was included.

**Figure 2:**
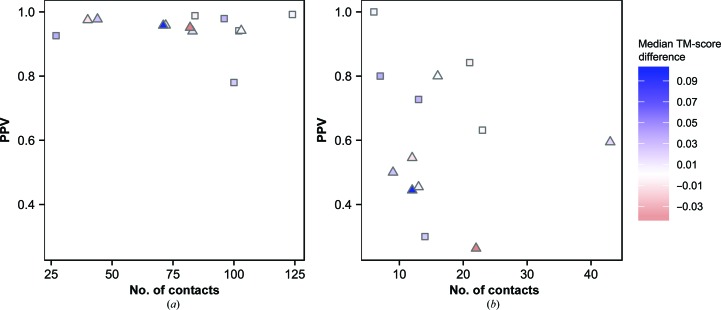
Effects of upweighting and addition of β-sheet-specific contacts on contact accuracy and decoy quality. Number of (*a*) upweighted and (*b*) added *bbcontacts* β-sheet-specific contact restraints for 13 β-sheet-containing (seven all-β, triangles; six mixed α–β, squares) targets plotted against their corresponding positive predictive value (PPV). The colour fill of each point corresponds to the resulting difference in median TM-scores between the largest cluster decoys from *PconsC*2-only and *PconsC*2+*bbcontacts* decoy sets (positive values favour the latter).

For 13 β-strand-containing proteins in the data set we developed a novel approach of combining the top-*L* predicted *PconsC*2 (Skwark *et al.*, 2014 ▸) contacts with the filtered β-sheet-specific *bbcontacts* (Andreani & Söding, 2015 ▸) contacts. This procedure resulted in the upweighting of some contacts already present in the *PconsC*2 list and the addition of others. The number of contacts affected in each category is shown in Fig. 2 ▸. At least 80% of upweighted contact restraints (present in the final contact lists of *PconsC*2 and *bbcontacts* predictions) proved to be true positives (Fig. 2 ▸ *a*). The average PPV of upweighted contacts was 95% (Fig. 2 ▸ *a*). The quality of the added contacts was generally lower (Fig. 2 ▸ *b*), but nine of the 13 targets had a PPV of at least 50% (Fig. 2 ▸ *b*). Thus, at the cost of the inclusion of some false-positive contacts, our approach generally provides extra valuable information for the folding process.

Models based on *PconsC*2+*bbcontacts* contacts were again somewhat improved compared with those built using the *PconsC*2 contacts: the median TM-scores for the two model sets were 0.522 and 0.506, respectively (Fig. 1 ▸ *b*). Model quality improved for nine targets, of which five showed improvements in median TM-score of at least 0.02 (Table 2 ▸). Model quality deteriorated for four targets, but for three of these the difference was very small: less than 0.02 (Table 2 ▸).

### 3.2. Contact-guided *ab initio* models extend the tractable target range of *AMPLE*   

With a demonstrable and significant improvement in decoy quality evident from the use of predicted contact restraints, the ability of *AMPLE* to solve the 21 protein targets using contact-guided decoys was then tested. For all targets, two sets of decoys were trialled deriving from *ab initio* structure prediction with no contact-prediction restraints or with *PconsC*2-only restraints. For β-strand-containing targets a third decoy set was created using *PconsC*2+*bbcontacts*-derived restraints. Structure solution of each target was attempted with these sets of structure predictions using default *AMPLE* methods. Successful MR was detected as previously by the ability of *SHELXE* main-chain tracing and density modification run on the MR placement to reach a correlation coefficient (CC) of ≥25 with a mean traced chain length of ≥10 residues (Sheldrick, 2010 ▸; Bibby *et al.*, 2012 ▸; Thorn & Sheldrick, 2013 ▸; Keegan *et al.*, 2015 ▸; Thomas *et al.*, 2015 ▸). As previously (Keegan *et al.*, 2015 ▸; Thomas *et al.*, 2015 ▸), we further required that an *R*_free_ value of ≤0.45 could be achieved after *ARP*/*wARP* (Cohen *et al.*, 2008 ▸) or *Buccaneer* (Cowtan, 2006 ▸) automatic rebuilding of the resulting *SHELXE* chain traces.

**Figure 3:**
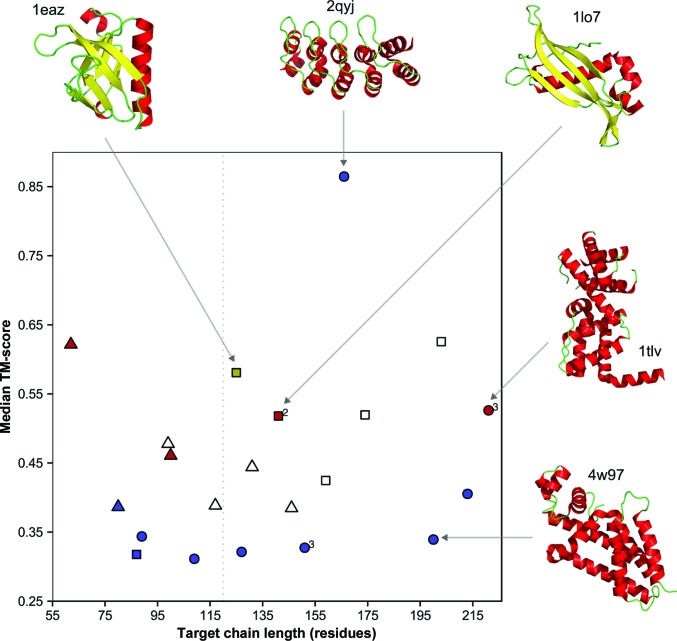
Contact restraint-guided *ab initio* models extend the tractable target range of *AMPLE*. Molecular-replacement (MR) success mapped against target chain length and median template-modelling score (TM-score). The point shape corresponds to the fold class of the target: all-α (circles), all-β (triangles) and mixed α–β (squares). The point colour indicates successful structure solutions for the contact constraints used: none (blue), *PconsC*2-only (red) and *PconsC*2+*bbcontacts* (gold). Points for successful solutions were considered in the order of *Rosetta*, *PconsC*2-only and *PconsC*2+*bbcontacts* decoys. In cases of unsuccessful molecular-replacement attempts (empty symbols), TM-scores for the largest clusters of *PconsC*2+*bbcontacts* decoys are shown. Median TM-scores for each point correspond to the largest decoy cluster (compared with the crystal structure), leading to a structure solution (cluster indices given next to each point for targets that were not solved with the largest cluster). The dashed grey line highlights the tested target chain-length limit of *AMPLE* (120 residues) for globular proteins (Bibby *et al.*, 2012 ▸). Cartoon representations of crystal structures of five different targets exemplify the diversity of structure solutions (PDB identifiers are provided next to each crystal structure). α-Helices are shown in red, β-sheets in yellow and loops in green.

Based on these stringent success criteria, the default algorithm of *AMPLE* achieved eight structure solutions for decoys predicted without contact restraints (Fig. 3 ▸, blue). Six out of eight all-α, one mixed α–β and one all-β target were solved with chain lengths up to 213 residues. Success using *ab initio* models has not been previously reported for such large globular protein targets (Table 3 ▸), but these findings recapitulate the fold-class preferences observed previously: *AMPLE* works well for all-α targets but less so for mixed α–β and particularly all-β proteins (Bibby *et al.*, 2012 ▸). Previously, the strong performance on all-α targets has been at least partly attributed to the greater accuracy of *Rosetta* modelling of those proteins (Bibby *et al.*, 2012 ▸), but these results make a second important contribution more explicit. Most all-α targets were solved despite the overall accuracy of their models being poor (Fig. 3 ▸). This suggests that their success in MR, nevertheless, lies with the superior ability of *SHELXE* to autotrace helices compared with other secondary structures (Sheldrick, 2010 ▸). The β-containing targets solved here were the mixed α/β-fold bacteriophage T4 glutaredoxin (PDB entry 1aba) and the all-β biotinyl domain of acetyl-coenzyme A carboxylase (PDB entry 1bdo), both with chain lengths of less than 90 residues (Table 3 ▸).

When predicted contact information from *PconsC*2 was used in the modelling, the resultant structure predictions from the largest cluster solved an additional two all-β structure solutions: the α-spectrin SH3 domain (PDB entry 2nuz) and the FN3con domain (PDB entry 4u3h) (Fig. 3 ▸, red). Although these targets do not exceed the previously benchmarked chain-size limit of 120 residues (Bibby *et al.*, 2012 ▸), it is worth noting that three out of the five all-β-containing proteins with chain lengths of less than 120 residues were solved. This strongly indicates that the previously low success rate of *AMPLE* of ∼2% for all-β targets in this size range (Bibby *et al.*, 2012 ▸) is improved by using contact information. *PconsC*2+*bbcontacts* decoys achieved all of the structure solutions using *PconsC*2-only decoys. Additionally and most notably, *PconsC*2+*bbcontacts* decoys led to the structure solution of the mixed α+β PH domain of the human TAPP1 protein (PDB entry 1eaz; Fig. 3 ▸, gold). The structure solution of 1eaz uniquely using *PconsC*2+*bbcontacts* restraints highlights the importance of the fusion of contact maps developed here. In total, the largest cluster decoys modelled with *PconsC*2 and *bbcontacts* restraints solved 11 out of 21 targets.

**Figure 4:**
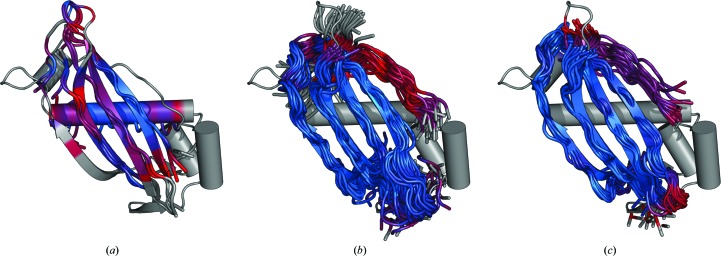
Contact restraints improve the search-model quality of β-strand-containing targets. Structural superposition of the (*a*) *Rosetta* (C^α^ r.m.s.d. 2.814 Å; ensemble contains two structures), (*b*) *PconsC*2-only (C^α^ r.m.s.d. 1.748 Å; 30 members) and (*c*) *PconsC*2+*bbcontacts* (C^α^ r.m.s.d. 1.760 Å; 15 members) search-model ensembles for 4-hydroxybenzoyl CoA thioesterase (PDB entry 1lo7). Examples are the highest scoring search models based on *SHELXE* CC score, with only (*b*) and (*c*) leading to successful structure solutions. Search models are shown as tubes and crystal structures as cartoons. (*a*) and (*c*) are 50% of the target sequence, while (*b*) is 55%. The colour scale illustrates the pairwise C^α^ r.m.s.d. between each search-model ensemble (represented by its first member) and the crystal structure, with blue representing the minimum C^α^ r.m.s.d. and red the maximum. Unaligned residues are coloured grey.

By default, the *AMPLE* algorithm processes *ab initio* models solely from the largest cluster. When trialling ensemble search models based on decoys from the three largest clusters, an additional three structure solutions were obtained (Fig. 3 ▸). The successful solution of haem-bound oxymyoglobin (PDB entry 1a6m) was achieved with *Rosetta* decoys from the third cluster. Notably, *ab initio* modelling of this target was performed without its large bound haem group, yet structure solution was achieved. The second target, the inactive LicT PRD domain from *Bacillus subtilis* (PDB entry 1tlv), was solved with *PconsC*2-only decoys from the third cluster. This all-α target with a chain length of 221 residues is, to our knowledge, the largest globular protein to be solved using search models derived from *ab initio* protein structure modelling. As mentioned above, all-α targets such as this benefit from the powerful helix tracing in *SHELXE* (Sheldrick, 2010 ▸), as do the programs of the *ARCIMBOLDO* suite (Rodríguez *et al.*, 2009 ▸), which can also solve large all-α protein structures (Fourati *et al.*, 2014 ▸). Lastly, *PconsC*2-only and *PconsC*2+*bbcontacts* decoys derived from second largest clusters yielded search models that solved the 4-hydroxy­benzoyl CoA thioesterase domain structure (PDB entry 1lo7). Particularly notable about this solution is the topology of the search models. Although this mixed α+β target contains a number of helices, the best structure solutions (based on *SHELXE* CC scores) were obtained from search models containing the accurately modelled, central, four-stranded β-sheet (Fig. 4 ▸). This accurate modelling, which is required for successful MR, was only achieved with the guidance of contact restraints. In total, the addition of these three structure solutions results in 14 out of 21 structure solutions for *PconsC*2+*bbcontacts* decoys compared with nine for simple *Rosetta* decoys.

**Figure 5:**
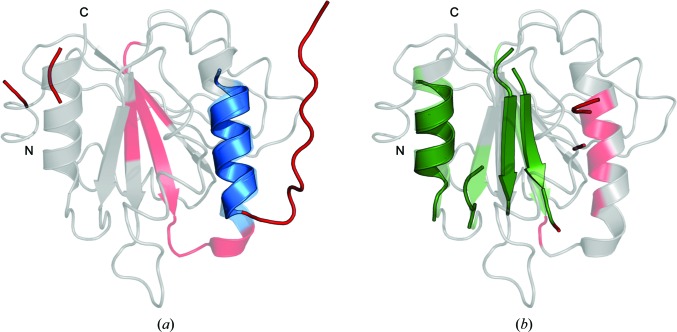
*PconsC*2+*bbcontacts* contact-derived models give a more accurate MR placement for PDB entry 1e0s which, although not solved automatically, might yield to expert manual intervention. Top *Phaser* solutions of 1e0s based on RIO scores for (*a*) *PconsC*2-only (RIO score 12) and (*b*) *PconsC*2+*bbcontacts* (RIO score 30) search models for target 1e0s. Search-model colour coding indicates useful superposition of residues by in-sequence (green) or out-of-sequence register (blue) residues as well as misplaced (red) residues. The addition of *bbcontacts* restraints produced a more accurate model with correctly placed β-strands that was placed correctly. Both structures are shown in cartoon representation with the crystal structure shown as a transparent cartoon. Unaligned reference crystal structure residues are coloured grey.

Although the stringent criteria of MR success used here did not indicate a successful structure solution for target 1e0s, the beneficial effect of including joint *PconsC*2+*bbcontacts* contact predictions was evident in the search-model placement as assessed by RIO scores (Fig. 5 ▸). For the top *PconsC*2-only search model, 40% (12 residues) of the search-model residues were correctly superimposed, albeit out of register (blue) on the target structure (*Phaser* TFZ = 4.7, *Phaser* LLG = 16). For the top *PconsC*2+*bbcontacts* search model, 77% (30 residues) of the search model were superimposed in an in-register fashion (*Phaser* TFZ = 5.3, *Phaser* LLG = 17) (Fig. 5 ▸). For the latter, expert manual intervention might allow structure determination, but in this case the correct solution was not prominent in the list of MR placements. Nevertheless, it is clear that even when overall structure solution was not automatically achieved the *PconsC*2+*bbcontacts* model provided better results which might be recoverable as successes in the future as MR and post-MR software improves still further.

Within the range explored, the success of structure solution did not appear to depend significantly on the resolution of the available crystallographic data (Supplementary Fig. S3). Successful targets ranged in resolution from 1.00 to 2.05 Å (mean ± standard deviation of 1.62 ± 0.32 Å), while unsuccessful targets spanned 1.04–2.28 Å (1.67 ± 0.40 Å). The solvent content of the protein crystals appeared to have a modest impact on MR success. Targets with successful structure solutions ranged from 36.0 to 55.3% (mean ± SD of 46.1 ± 5.2%) solvent content compared with 25.8–48.0% (39.1 ± 8.1%) for unsuccessful targets.

**Figure 6:**
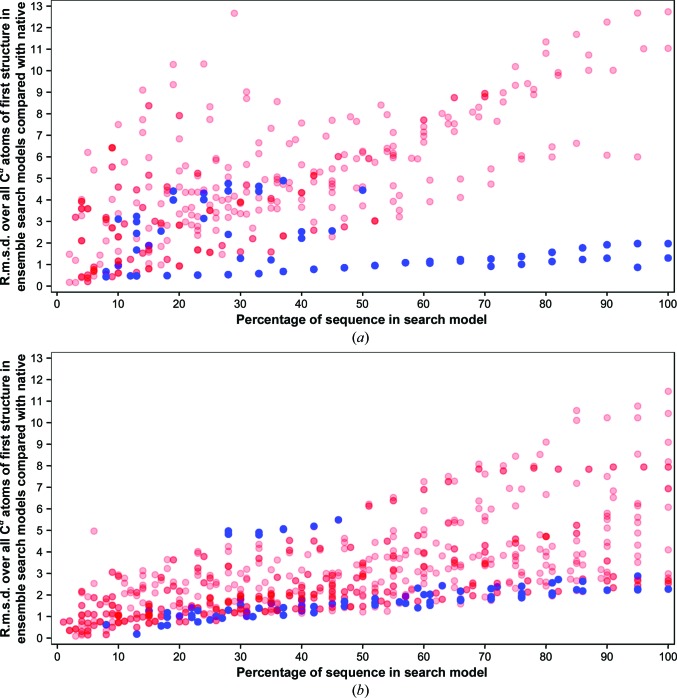
Variance-based truncation remains an effective way to derive successful search models from higher quality contact restraint-assisted *ab initio* decoys. The percentage of sequence in the search model is mapped against the root-mean-square deviation (r.m.s.d.) over all C^α^ atoms of the first representative of each search-model ensemble derived from the largest cluster against the native structure. Successful structure solutions of individual search models are highlighted in blue and unsuccessful solutions in red. Progressively darker shades of either colour correspond to increasing numbers of overlapping points. Progressive truncation is shown for (*a*) *Rosetta* decoys and (*b*) *PconsC*2+*bbcontacts* decoys (or *PconsC*2-only decoys for all-α targets).

Given that the inclusion of predicted contact information is a significant change to the modelling protocol, we re-examined the performance and importance of the key features of the operation of *AMPLE*. A detailed analysis of the characteristics of the successful search models is provided in the Supporting Information; only a summary is provided here. *AMPLE* uses well established clustering of decoys (Simons *et al.*, 1997 ▸) to pick out those likely to be the most accurate. This continues to be effective here, as picking the largest clusters selects better than average decoys from the sets available (Supplementary Fig. S4) and there is a good correlation between the largest cluster size and the median TM-score of the decoys in that cluster (Supplementary Fig. S5). However, the size of the largest cluster does not correlate well with the total number of successful search models (Supplementary Fig. S6). *AMPLE* also relies on rational, variance-based truncation to trim ensembles down to more accurate core structures, with the size range 15–40 residues found to be most successful (Bibby *et al.*, 2012 ▸). Here, the truncation is further validated (Fig. 6 ▸) and a similar mapping of success onto search-model size is observed (Supplementary Fig. S7). Fig. 6 ▸ further illustrates the overall positive impact of contacts on accuracy: note the larger number of low-r.m.s.d. ensembles on the right of Fig. 6 ▸(*b*) compared with Fig. 6 ▸(*a*). However, Fig. 6 ▸ also illustrates that targets that are already well modelled by simple *Rosetta* and successful in MR (blue points on the right in Fig. 6 ▸ *a*) can be modelled slightly worse when contact information is included (somewhat raised r.m.s.d.s in Fig. 6 ▸ *b*), presumably owing to the influence of false-positive contact predictions. Previously, we have found that sampling across three subclustering radii and three modes of side-chain treatment were both required for solution of the largest possible number of targets (Bibby *et al.*, 2012 ▸). This remains largely the case in the current exercise, as unique solutions were obtained for each of the sub­clustering radii (Supplementary Table S1). Polyalanine side-chain search models were the most successful, but a single target, PDB entry 1eaz, was only solved using one of the alternative treatments (Supplementary Table S1).

## 4. Discussion   

The recently emerged ability to predict contacting residues from large protein sequence alignments is one of the most exciting developments in structural bioinformatics for many years. The key statistical breakthrough allowing the disentangling of predicted direct contacts (Giraud *et al.*, 1999 ▸; Miller & Eisenberg, 2008 ▸; Weigt *et al.*, 2009 ▸; Marks *et al.*, 2011 ▸), *i.e.* neighbouring amino acids from pairs of residues whose identities covary indirectly, has been followed by a wave of papers not only dealing with the accuracy of predictions but also considering the manifold applications of the information. Predicted contact information is of immediate benefit to crystallographers in many ways that are yet to be fully appreciated, including parsing of domains for structural analysis (Rigden, 2002 ▸; Sadowski, 2013 ▸) and interpretation of crystal structure composition (Nicoludis *et al.*, 2015 ▸). Here, we considered how the better protein *ab initio* models that can be produced by exploiting information can serve as a source of improved search models for MR. We use the MR pipeline *AMPLE* as a convenient and effective tool for the analysis.

Challenged by the lesser success of *AMPLE* with β-structure-containing proteins (Bibby *et al.*, 2012 ▸), and motivated by the accuracy improvements in *ab initio* fold predictions through contact restraints (Marks *et al.*, 2011 ▸; Michel *et al.*, 2014 ▸; Jones *et al.*, 2015 ▸; Ovchinnikov *et al.*, 2015 ▸), we developed a new approach for combining predicted contact-restraint lists from *PconsC*2 (Skwark *et al.*, 2014 ▸) and *bbcontacts* (Andreani & Söding, 2015 ▸) to elevate the *ab initio* modelling accuracy of β-structure protein targets. Structure predictions guided by the resulting *PconsC*2+*bbcontacts* contact restraints improved the decoy quality for nine out of 13 β-structure-containing protein targets. Our approach, which involved both selective upweighting of and addition to the *PconsC*2 set, based on the specialist β-sheet predictions, may well be of more general use to the protein-modelling community. The value of these contact-guided *ab initio* models for structure solution by MR of targets treated as novel folds is demonstrated. Nine of the 21 targets in the data set were solved using the *AMPLE* algorithm to process unassisted *Rosetta* structure predictions. This number rose to 14 using contact-guided modelling. The 100% success rate for all-α targets is highly encouraging and, along with the comparable MR pipeline *ARCIMBOLDO* (Rodríguez *et al.*, 2009 ▸), graphically illustrates the power of the α-helical tracing implemented in *SHELXE* (Sheldrick, 2010 ▸) and the relative tractability of α-rich targets to unconventional MR. More β-rich, α-poor targets are harder for both *AMPLE* and *ARCIMBOLDO*, so our demonstrable advances with these targets, leveraging the value of contact restraints during *ab initio* modelling, are exciting. The fusion of top-ranked *PconsC*2 and *bbcontacts* contacts developed as part of this study proved to be a key part in one successful structure solution, further highlighting the importance of the approach.

The size of the targets solved is another notable feature of this work. We originally suggested (Bibby *et al.*, 2012 ▸) that all-α protein targets larger than the 120-residue threshold then tested could be suitable for the cluster-and-truncate approach of *AMPLE*. Here, we demonstrate this to be true, with unassisted decoys leading to the solution of a 213-residue protein and contact-assisted models leading to the successful solution of a 221-residue chain. To our knowledge, these are the largest targets to be solved with search models derived from *ab initio* structure decoys.

The availability of reliable contact restraints to aid MR with *ab initio* models clearly widens the range of targets for which *AMPLE* is a viable option for structure solution. The accuracy of contact predictions is directly related to the number of protein sequences deposited in sequence databases such as UniProt (The UniProt Consortium, 2015 ▸), and thus will benefit from the continuous growth of those databases. Notably, this manuscript focused solely on globular proteins; yet the *AMPLE* algorithm is equally well suited to coiled-coil and transmembrane proteins (Thomas *et al.*, 2015 ▸ and unpublished data). Specific contact predictors for the latter are available (Wang *et al.*, 2011 ▸; Hopf *et al.*, 2012 ▸; Yang *et al.*, 2013 ▸; Zhang *et al.*, 2016 ▸) and future research will explore their application to MR using *AMPLE*. In conclusion, the current and future broadening of the target range tractable by *AMPLE* through the use of evolutionary restraints during *ab initio* modelling highlights the value of the software as an effective alternative to experimental phasing approaches in X-ray crystallography.

In summary, we confirm here that predicted contacts can significantly improve *ab initio* model quality in a way that directly impacts on structure solution by MR. Our novel mode of uniting general and β-structure-specific contact predictions brings further tangible model improvements to the particularly difficult β-rich protein targets. All of these methodo­logical advances have immediate benefits for crystallographers facing targets with novel or divergent folds which cannot be addressed by conventional MR. *AMPLE* proves to be an efficient framework for rendering these contact-assisted decoys into search-model ensembles, with truncation and extensive sampling remaining key to success. Future inevitable expansion of sequence databases and predictable improvements in contact-prediction software will undoubtedly extend the reach of MR with *ab initio* models still further.

## Supplementary Material

Supplementary Table S1.. DOI: 10.1107/S2052252516008113/lz5010sup1.xlsx

Supporting information including Supplementary Figures S1-S8.. DOI: 10.1107/S2052252516008113/lz5010sup2.pdf

## Figures and Tables

**Table 1:** Restraint-guided *ab initio* modelling improves model quality Median template-modelling scores for *ab initio* decoys found in the largest cluster (values for all decoys are shown in parentheses) predicted for threefold classes using three different types of residue–residue constraint settings.

Fold classification	*Rosetta*	*PconsC*2-only	*PconsC*2+*bbcontacts*
All-α	0.377 (0.298)	0.609 (0.531)	—
Mixed α–β	0.314 (0.252)	0.537 (0.433)	0.565 (0.441)
All-β	0.323 (0.247)	0.467 (0.374)	0.471 (0.381)
Mixed α–β + all-β	0.320 (0.249)	0.506 (0.397)	0.522 (0.403)

**Table 2:** Summary of *ab initio* structure-prediction results of β-structure-containing targets All data shown are for *PconsC*2+*bbcontacts* (*PconsC*2-only) guided decoys.

					TM-score	
Fold classification	PDB code	No. of effective sequences	No. of contacts	PPV	Top-cluster decoys	1000 decoys
Mixed α–β	1aba	1037	92 (87)	0.787 (0.782)	0.584 (0.546)	0.507 (0.496)
	1chd	852	222 (203)	0.924 (0.931)	0.626 (0.634)	0.501 (0.528)
	1e0s	1831	184 (174)	0.691 (0.713)	0.520 (0.495)	0.362 (0.353)
	1eaz	1060	136 (125)	0.928 (0.944)	0.581 (0.547)	0.512 (0.460)
	1lo7	1026	146 (141)	0.980 (0.986)	0.535 (0.528)	0.453 (0.443)
	1tjx	1189	178 (159)	0.857 (0.887)	0.425 (0.427)	0.354 (0.358)
All-β	1bdo	940	91 (80)	0.913 (0.963)	0.477 (0.490)	0.379 (0.402)
	1kjl	272	183 (146)	0.704 (0.727)	0.385 (0.367)	0.313 (0.293)
	1npu	943	136 (117)	0.835 (0.940)	0.389 (0.438)	0.322 (0.331)
	1pnc	887	111 (99)	0.830 (0.889)	0.478 (0.470)	0.375 (0.354)
	2nuz	1048	70 (62)	0.901 (0.952)	0.650 (0.622)	0.540 (0.498)
	3w56	949	146 (131)	0.896 (0.906)	0.444 (0.440)	0.363 (0.353)
	4u3h	1226	109 (100)	0.911 (0.950)	0.563 (0.461)	0.440 (0.446)

**Table 3:** Summary of molecular-replacement solutions of 21 protein targets The total number of ensemble search models derived from *ab initio* decoys from the three largest clusters is provided in parentheses after the individual number of successful search models.

				No. of successful (total) search models		
Fold classification	PDB code	Resolution (Å)	Target chain length	*Rosetta*	*PconsC*2-only	*PconsC*2+*bbcontacts*
All-α	1kw4	1.75	89	137 (393)	101 (468)	—
	1bkr	1.10	109	21 (105)	13 (459)	—
	4cl9	1.40	127	1 (210)	1 (408)	—
	1a6m	1.00	151	1 (102)	4 (327)	—
	2qyj	2.05	166	378 (501)	329 (453)	—
	4w97	1.60	200	6 (114)	3 (399)	—
	1hh8	1.80	213	3 (66)	0 (297)	—
	1tlv	1.95	221	0 (18)	2 (399)	—
Mixed α–β	1aba	1.45	87	4 (312)	58 (429)	93 (411)
	1eaz	1.40	125	0 (135)	0 (345)	28 (327)
	1lo7	1.50	141	0 (120)	3 (327)	3 (333)
	1tjx	1.04	159	0 (27)	0 (165)	0 (150)
	1e0s	2.28	174	0 (15)	0 (195)	0 (207)
	1chd	1.75	203	0 (12)	0 (279)	0 (225)
All-β	2nuz	1.85	62	0 (393)	76 (444)	183 (453)
	1bdo	1.80	80	27 (343)	16 (381)	19 (372)
	1pnc	1.60	99	0 (126)	0 (300)	0 (297)
	4u3h	1.98	100	0 (273)	14 (357)	1 (372)
	1npu	2.00	117	0 (111)	0 (210)	0 (180)
	3w56	1.60	131	0 (129)	0 (123)	0 (150)
	1kjl	1.40	146	0 (63)	0 (174)	0 (201)
