# Supplementary material for: Residue contacts predicted by evolutionary covariance extend the application of ab initio molecular replacement to larger and more challenging protein folds
Source: IUCrJ. 2016 Jun 15;3(Pt 4):259–70. doi: 10.1107/S2052252516008113 (PMC4937781; doi:10.1107/S2052252516008113)

IUCrJ

Volume 3 (2016)

Supporting information for article:

Residue contacts predicted by evolutionary covariance extend the application of *ab initio* molecular replacement to larger and more challenging protein folds

Felix Simkovic, Jens M. H. Thomas, Ronan M. Keegan, Martyn D. Winn, Olga Mayans and Daniel J. Rigden

S1. Characteristics of successful search models

With much improved decoy quality deriving from the use of predicted residue restraints to guide *ab initio* structure prediction, the question arises whether AMPLE's existing cluster-and-truncate approach remains the most suitable for obtaining a conserved, native-like core from the decoys found in the largest clusters. For globular targets solved using simple Rosetta decoys, certain features throughout AMPLE's cluster-and-truncate approach typically correlated with eventual success in structure solution (Bibby et al., 2012). In general, the greater the number of decoys in the largest cluster the more likely the success was with derived search models. Truncation removed structurally variant parts leading to smaller more accurate ensemble subsets of the cluster decoys. Although successful search models were found at every truncation interval, the majority were derived with search models containing around 30 residues. Lastly, each of the potential nine search models derived at each truncation level (three subclustering radii with three side chain treatments each) can lead to non-redundant structure solutions. Similar observations, particularly with respect to the most successful search model size range were made for other target classes (Thomas et al., 2015; unpublished data) and for *ab initio* models made with QUARK (Keegan et al., 2015).

A size comparison of the largest clusters of Rosetta and PconsC2+bbcontacts (or PconsC2-only for all-α) decoys indicated a median increase of 122 decoys per cluster in the latter. All cluster sizes increased except for target 2qyj. More accurate *ab initio* models are directly linked to larger cluster sizes because of the associated increase in convergence (Simons et al., 1997). Here, as expected, the largest cluster contains better than average quality decoys (*Figure S4*) but the size of the largest cluster does not link to the total number of successful search models (*Figure S6*).

In comparison to the clustering step, the progressive truncation of decoys in the largest cluster at 20 different intervals directly affects the number of successful search models. An analysis of the progressive truncation and the effects on search model accuracy revealed that all successful search model ensembles had a Cα-rmsd better than 5.5Å compared to the native structure (*Figure 6*). Although the latter cutoff is independent of whether contact information was provided during *ab initio*

modelling, a clear difference between the Rosetta and PconsC2+bbcontacts (or PconsC2-only for all-α) ensemble search models for all targets can be observed. In total, Rosetta decoys for all targets produced 1314 ensemble search models based on the largest clusters. In comparison, PconsC2+bbcontacts decoys generated for the same targets 2469 search model ensembles from the largest clusters. This increase is the result of a more successful subclustering process due to the increased structural homogeneity across the decoys in the largest cluster. The most notable difference between the two sets is detected for the Small G-protein ARF6-GDP (PDB: 1e0s), which produced 3 ensemble search models based on Rosetta decoys and 90 based on PconsC2+bbcontacts decoys. Additionally, ensemble search models with structural fragments of 15-40 residues of the target sequence are more likely to succeed in MR phasing than larger or smaller search models (Bibby et al., 2012). Here we find that the same range is most successful for contact-guided decoys (*Figure S7*). Out of 246 successful search models for PconsC2+bbcontacts decoys derived from the largest cluster (PconsC2-only for all-α), 101 successful search models contained 15-40 residues. Significantly, some cases like the PH domain of TAPP1 (PDB: 1eaz) and the N-terminal bromodomain of human BRD4 (PDB: 4cl9) only solved with truncated search models in this size range. Nevertheless, structure solutions were also achieved with larger or smaller search models. The smallest search model leading to a structure solution contained nine residues (8% of total sequence) and solved the Calponin homology (CH) domain from human Beta-spectrin (PDB: 1bkr). In comparison, the largest successful search model in terms of residues was found for the designed full consensus ankyrin (PDB: 2qyj) domain with 158 residues (95% of total), and in terms of percentage of the total sequence the untruncated, 62 residue search model for α-spectrin SH3 domain (PDB: 2nuz) was successful (*Figure S8*). Therefore, although truncating the *ab initio* models at different levels remains essential for contact-guided decoys, biasing sampling into the most successful size range may be advantageous in future runs.

The truncated decoys are further processed by subclustering at three different atomic radii, with the resulting subclusters previously found to be similarly successful (Bibby et al., 2012). Similar trends are seen here: 36% of structure solutions with Rosetta decoys were achieved with a subclustering

radius of 1Å, 36% at a radius of 2Å, and 28% at a radius of 3Å (*Supplementary Table*). For PconsC2+bbcontacts (or PconsC2-only for all-α) decoy sets similar numbers were observed (35% at radius of 1Å; 40% at 2Å; 25% at 3Å). Nevertheless, in terms of number of targets solved all three subclustering radii were essential. Largest-cluster decoys for target 1eaz produced a total of 327 search models, but only one solved and this derived from a subclustering radius of 1Å. In comparison, contact-guided decoys from the largest cluster for target 4u3h achieved structure solutions solely with decoys subclustered at 2Å. A single search model with subclustering radius of 3Å solved the target 4cl9 with Rosetta decoys.

The final step in search model creation is the side-chain processing of each subclustered ensemble. Similarly to the subclustering, no difference was observed between Rosetta and PconsC2+bbcontacts decoys (*Supplementary Table*). For both the polyalanine treatment is most successful, covering 37% of successful search models for Rosetta decoys and 44% for PconsC2+bbcontacts decoys. For almost all targets, the polyalanine side-chain treatment would be enough to obtain a structure solution. However, some cases, like the target 1eaz, only solve with either or both of the remaining treatments. Thus, relying solely on polyalanine side-chain treatment may limit the overall success rate, although trialling polyalanine ensemble search models first might lead to structure solution faster.

Figure S1 Flowchart illustrating the steps involved in residue-residue coupling prediction to guide *ab initio* structure prediction. The three major steps are highlighted in italic font. Figure adapted from Marks et al. (2012).

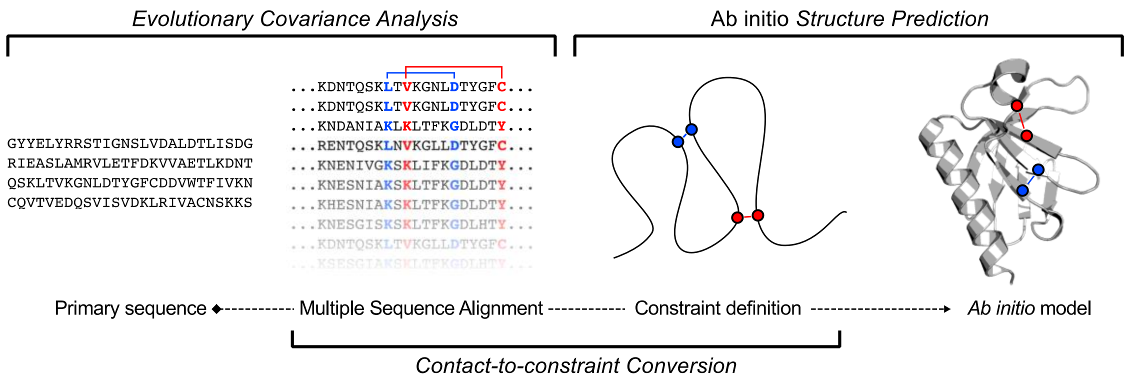

Figure S2 The number of effective sequences influences the accuracy of contact predictions. Number of effective sequences plotted against the positive predictive value (PPV) of (a) top-L PconsC2-only and (b) top-L PconsC2 plus bbcontacts contact lists. Symbol fills correspond to the median TM-scores for top-cluster decoys predicted with the corresponding contact lists. Symbol shapes correspond to the three different fold classes: all-α (circle), mixed α-β (square), and all-β (triangle).

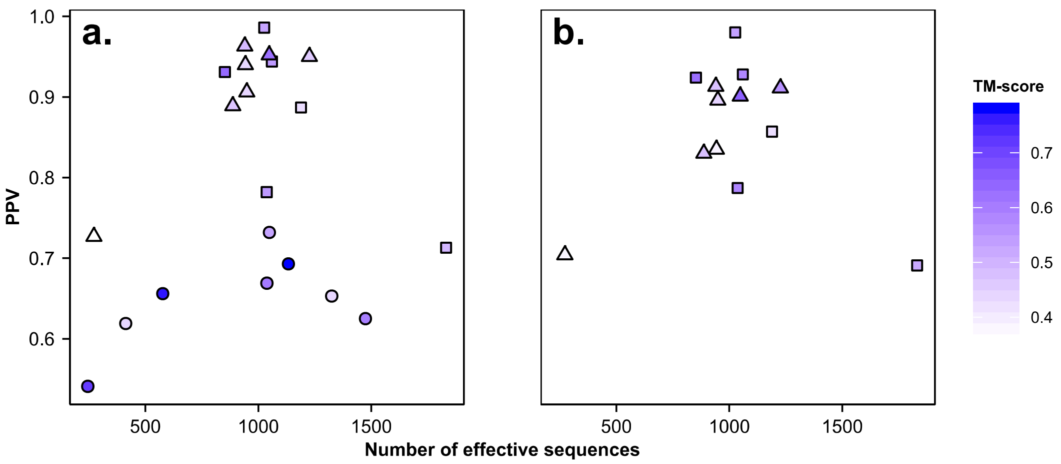

Figure S3 Resolution and solvent content have no apparent effect on the likelihood of structure solution. Molecular Replacement (MR) success mapped against target chain length and (a) resolution or (b) solvent content. The point shape corresponds to the fold class of the target: all-α (circle), all-β (triangle), and mixed α-β (square). The point colour indicates successful structure solutions for the contact constraints used: none (blue), PconsC2-only (red) and PconsC2+bbcontacts (gold). Points for successful solutions were considered in the order of Rosetta, PconsC2-only, and PconsC2+bbcontacts decoys. In cases of unsuccessful Molecular Replacement attempts empty symbols are shown, Numbers beside points indicate successful solutions derived from clusters other than the largest.

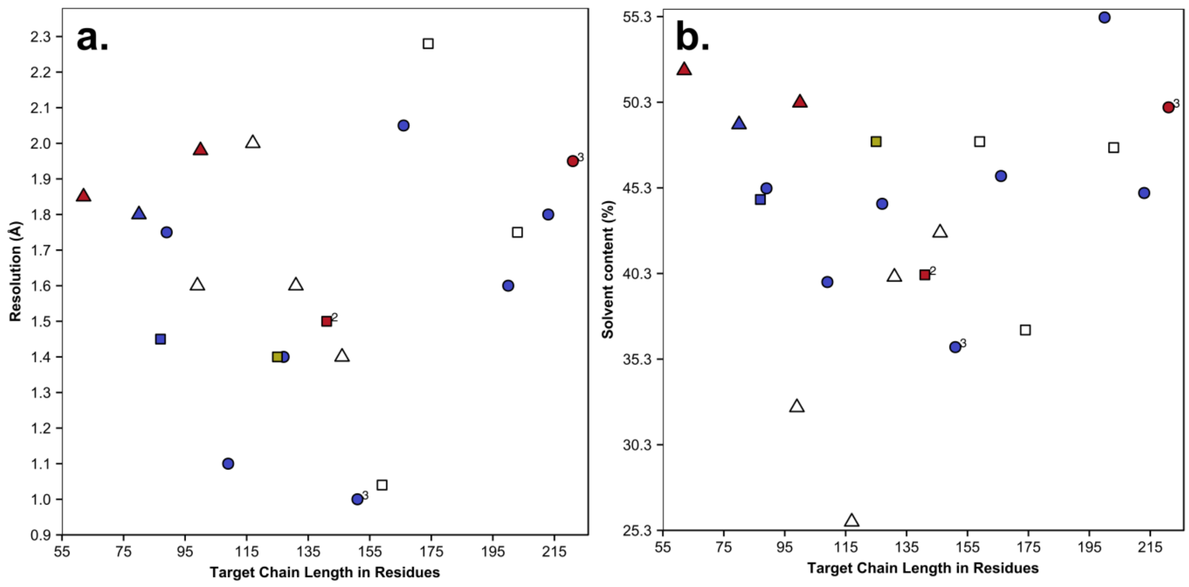

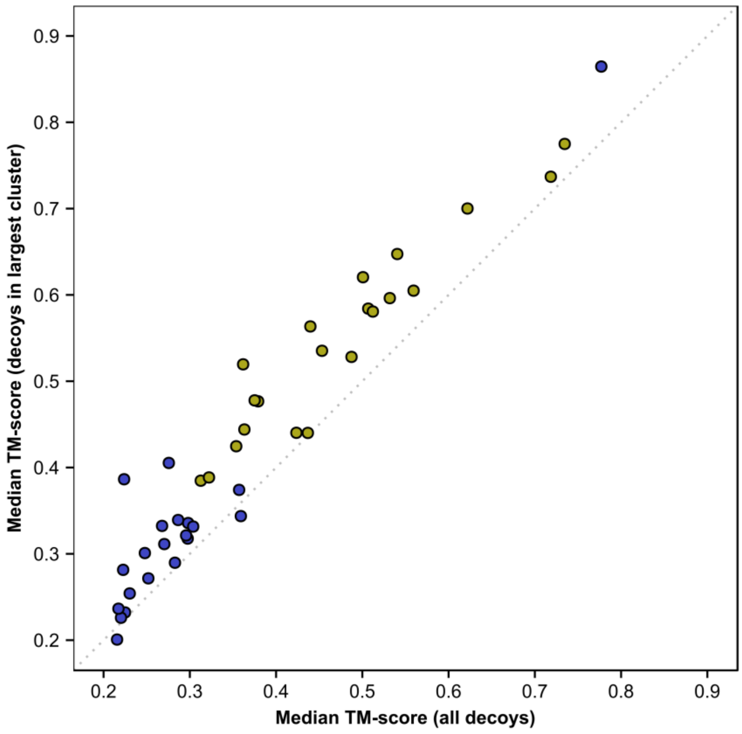

Figure S4 Clustering allows for the subselection of better than average decoys. Median TM-scores for all decoys are plotted against the median TM-scores for decoys found in the largest cluster for Rosetta (blue) and PconsC2+bbcontacts (PconsC2-only decoys for all-α targets) (gold) decoys.

Figure S5 Decoy quality is directly linked to the size of decoy clusters. The number of decoys found in the largest cluster is mapped against the corresponding median TM-score for Rosetta (blue) and PconsC2+bbcontacts (PconsC2-only decoys for all-α targets) (gold) decoys.

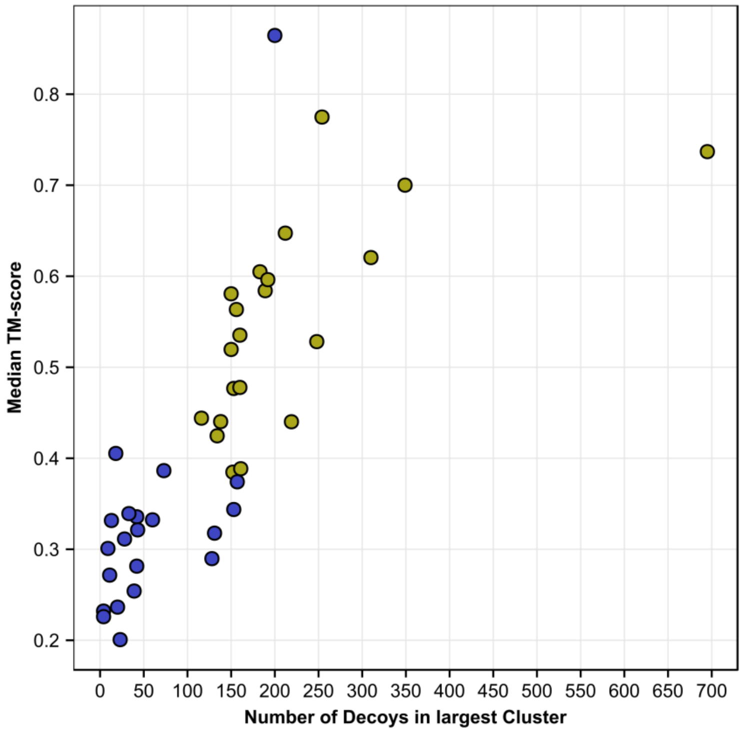

Figure S6 Larger clusters are associated with a higher number successful search models. Number of decoys in the largest cluster mapped against the number of search model ensembles leading to structure solution for Rosetta (blue) and PconsC2+bbcontacts (PconsC2-only decoys for all-α targets) (gold) decoys.

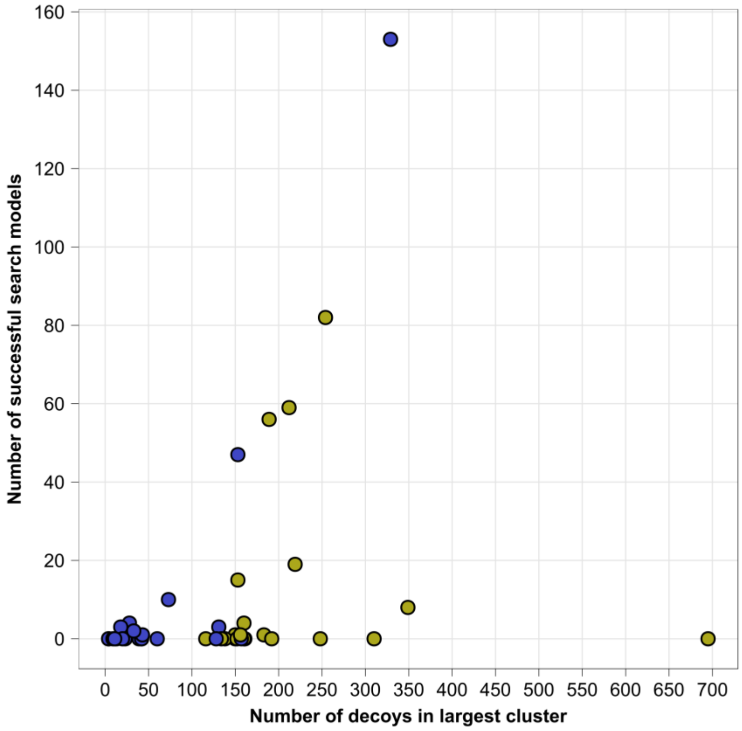

Figure S7 Smaller search models lead to structure solution more often. (a) Percentage of residues and (b) number of residues per chain in search model mapped against the number of search models leading to structure solution (blue) or not (red).

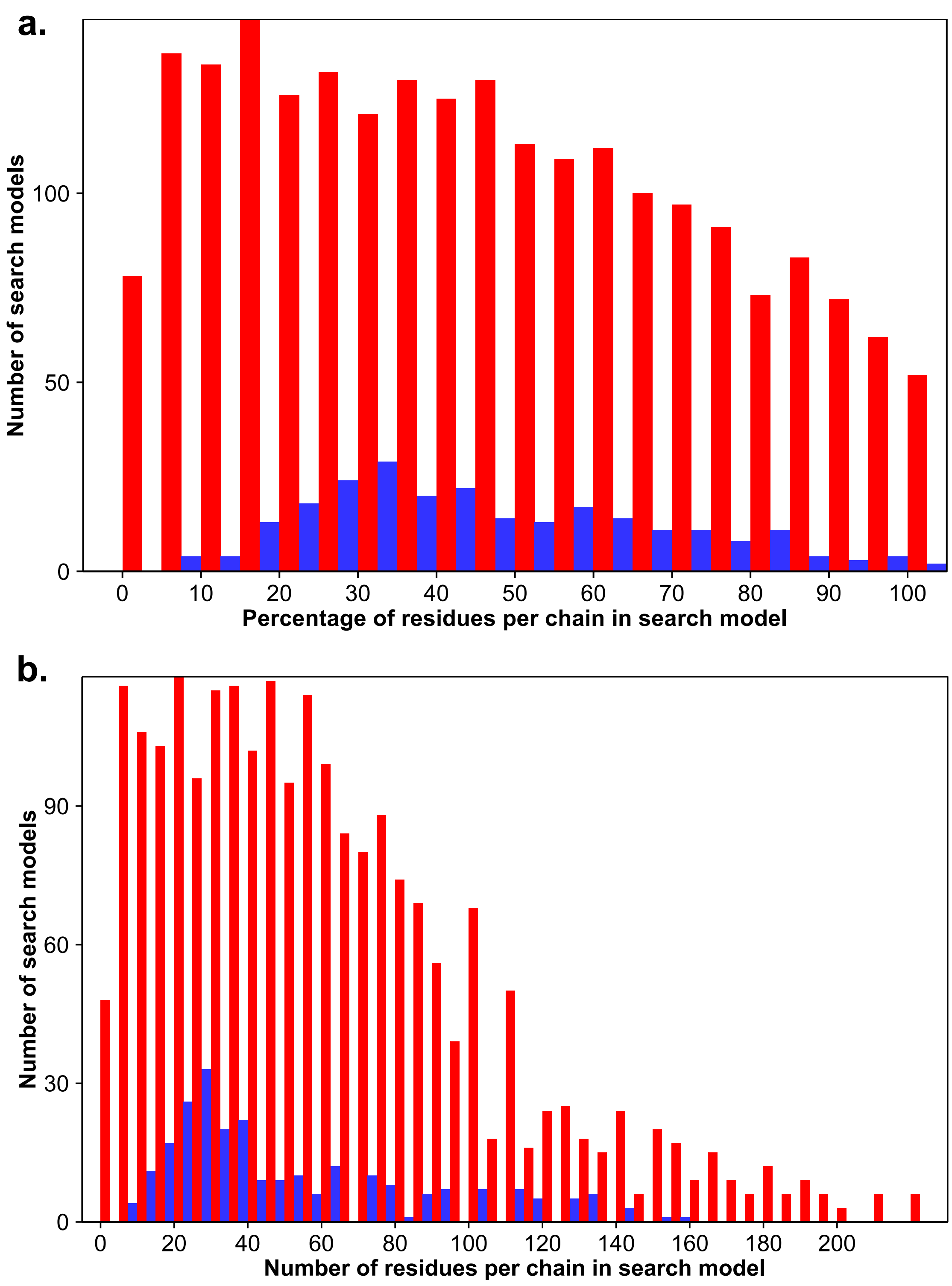

Figure S8 Successful search models are found across a great variety of truncation levels and side-chain treatments. Different truncation levels exemplified by a range of different-sized PconsC2+bbcontacts (PconsC2-only for all-α) search models leading to structure solution: (a) 9 residue fragment (8%) for target 1bkr (Cα-rmsd: 1.916 Å; RIO out-of-sequence register: 6); (b) 23 residue fragment (18%) for target 1eaz (Cα-rmsd: 2.053 Å; RIO in-sequence register: 17); (c) 141 residue fragment (95%) for target 2qyj (Cα-rmsd: 2.241 Å; RIO in-sequence register: 61); (d) 62 residues (100%) for target 2nuz (Cα-rmsd: 1.163 Å; RIO in-sequence register: 20). All targets shown in gray, all ensemble search models in green.

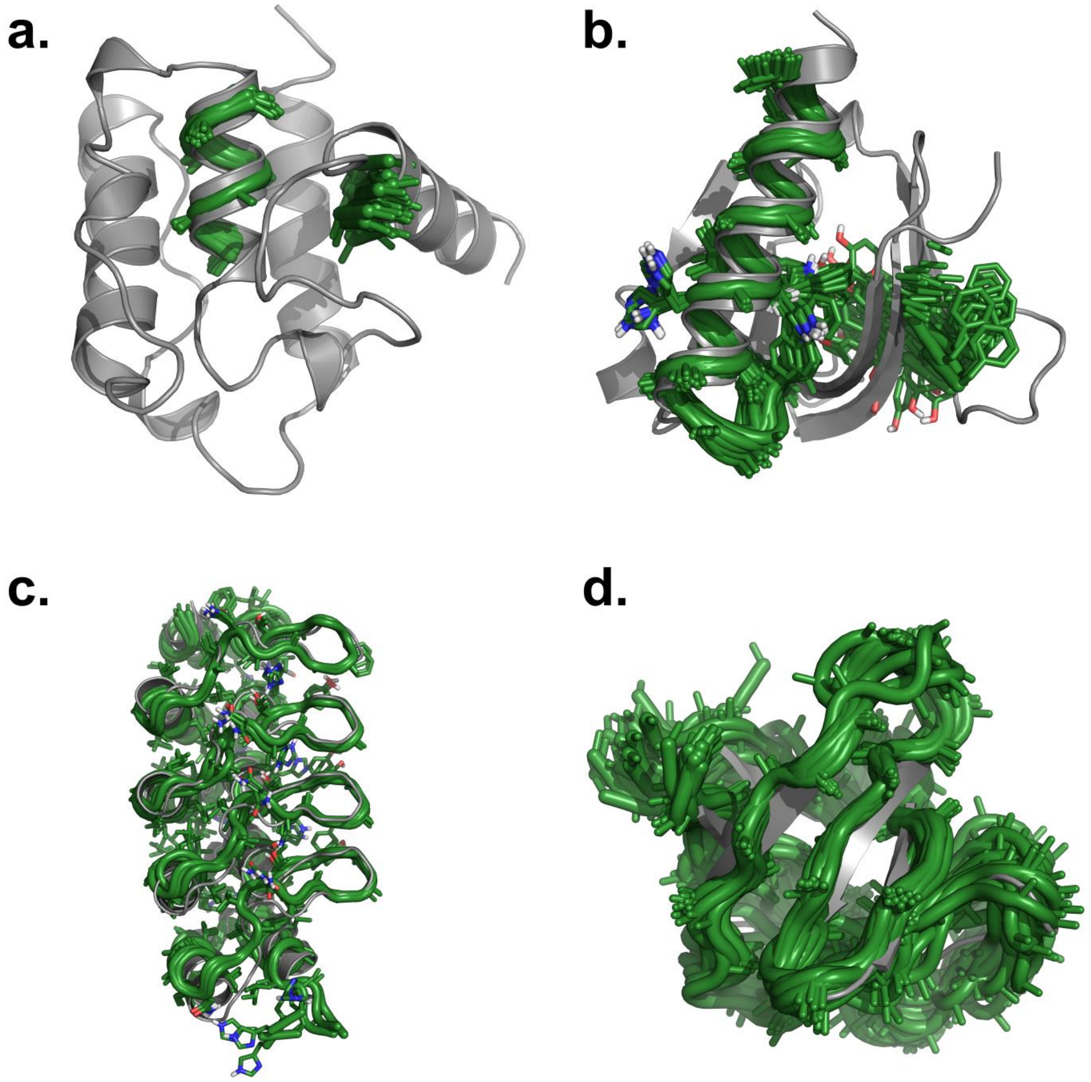

Supplement: Supplementary file 2 [file m-03-00259-sup2.pdf]
